# Discovery of Fragment Molecules That Bind the Human Peroxiredoxin 5 Active Site

**DOI:** 10.1371/journal.pone.0009744

**Published:** 2010-03-17

**Authors:** Sarah Barelier, Dominique Linard, Julien Pons, André Clippe, Bernard Knoops, Jean-Marc Lancelin, Isabelle Krimm

**Affiliations:** 1 Laboratory of Analytical Sciences (UMR CNRS 5180), Université Claude Bernard - Lyon 1, Bât. ESCPE Lyon, Domaine scientifique de la Doua, Villeurbanne, France; 2 Laboratory of Cell Biology, Institut des Sciences de la Vie, Université catholique de Louvain, Louvain-la-Neuve, Belgium; CNRS, France

## Abstract

The search for protein ligands is a crucial step in the inhibitor design process. Fragment screening represents an interesting method to rapidly find lead molecules, as it enables the exploration of a larger portion of the chemical space with a smaller number of compounds as compared to screening based on drug-sized molecules. Moreover, fragment screening usually leads to hit molecules that form few but optimal interactions with the target, thus displaying high ligand efficiencies. Here we report the screening of a homemade library composed of 200 highly diverse fragments against the human Peroxiredoxin 5 protein. Peroxiredoxins compose a family of peroxidases that share the ability to reduce peroxides through a conserved cysteine. The three-dimensional structures of these enzymes ubiquitously found throughout evolution have been extensively studied, however, their biological functions are still not well understood and to date few inhibitors have been discovered against these enzymes. Six fragments from the library were shown to bind to the Peroxiredoxin 5 active site and ligand-induced chemical shift changes were used to drive the docking of these small molecules into the protein structure. The orientation of the fragments in the binding pocket was confirmed by the study of fragment homologues, highlighting the role of hydroxyl functions that hang the ligands to the Peroxiredoxin 5 protein. Among the hit fragments, the small catechol molecule was shown to significantly inhibit Peroxiredoxin 5 activity in a thioredoxin peroxidase assay. This study reports novel data about the ligand-Peroxiredoxin interactions that will help considerably the development of potential Peroxiredoxin inhibitors.

## Introduction

The search for new protein ligands is central to chemical biology and drug discovery. The fragment-based approach represents an interesting method that combines random screening and rational structure-based design for the elaboration of bioactive compounds [1]–[4]. The method proposed a decade ago by the Abbott Company to design drugs and inhibitors consists in screening simple molecules, called the fragments, mostly defined by their weak molecular weight (<300 Da) [2], [3]. Due to the low complexity of the fragments, the binding affinity of the fragment-protein complexes are weak, usually in the mM order, in contrast with the µM activity generally achieved with molecules resulting from High Throughput Screening [2]. However, screening drug-sized molecules tends to favor ligands with several sub-optimal interactions, whereas fragments were shown to exhibit more favorable binding energies relative to their molecular size, leading to higher ligand efficiencies [5], [6]. As largely demonstrated in the literature, chemical modifications of these initial fragment hits can yield very potent molecules [2], [4], [7], [8].

The identification of initial fragments is a critical step for the success of the approach. In order to validate a fragment as an interesting starting point, its binding site, binding mode and binding affinity have to be characterized. Due to the low stability of the complex, the binding mode of the fragment is particularly challenging to determine. If X-Ray crystallography represents a very powerful tool for the resolution of protein-fragment costructures [9], the method requires high-quality crystals and highly soluble fragment molecules. Nuclear Magnetic Resonance (NMR) represents an alternative method where modifications of NMR parameters upon ligand binding are used to calculate 3D structures of the complexes [10]–[12]. However, these methods can be time-consuming and may be inappropriate for weak and small ligands such as fragments. Recently, the combination of NMR data such as ligand-induced chemical shifts and docking calculations was proposed to rapidly and efficiently obtain information about the binding site and binding mode of ligands [13]–[15].

Our study focuses on the interactions of fragment molecules with Peroxiredoxins. Peroxiredoxins (PRDXs) constitute a superfamily of enzymes that catalyze the reduction of hydroperoxides through a conserved catalytic peroxidatic cysteine (Cp) [16]–[18]. The members of the PRDX family share a highly conserved core structure derived from the thioredoxin fold, but display important variations in quaternary structures. If the structural properties of these proteins have been extensively studied, the enzymatic properties and the substrate specificities are less well characterized [18]–[24]. The design of PRDX inhibitors should provide important information for the understanding of the distinct biological roles of the PRDX enzymes. To date, few inhibitors or active site ligands have been discovered for these enzymes. The benzoate was reported as a ligand of the human Peroxiredoxin 5 protein (PRDX5), as it was observed in the crystal structure of the protein [25]. However, no information was published about its affinity towards the enzyme. More recently, a covalent inhibitor of the Peroxiredoxin TgPrxII from the parasite *Toxoplasma gondii* was published [26].

Here we report for the first time the screening of a homemade library composed of 200 fragments using ligand-observed NMR methods on the recombinant human PRDX5 protein. The backbone NMR resonances of the reduced active form of PRDX5 have been assigned, and ligand-induced chemical shift changes were used to drive the docking of the molecules into the protein structure. Both the binding site and the orientation of the molecule in the binding pocket were determined for the hit fragments and were confirmed by the study of fragment homologues. Also, the hit fragment F152 (catechol) and its homologue, compound D3 (4-methyl-catechol) were shown to inhibit the thioredoxin peroxidase activity of the enzyme. This work presents novel data about the ligand-PRDX interactions, and the molecules identified here are shown to represent interesting starting points for lead generation against PRDX5.

## Results

### Elaboration of a fragment library

We previously designed a small generalist library of 60 fragments and used it to elaborate an inhibitor of the Creatine kinase protein [27], based on the identification of an initial fragment by NMR screening. Here, the library was enlarged and contains 200 fragments that correspond to small commercial compounds with physicochemical properties defined by the rule of three [28]. The 140 newly added fragments were chosen so as to enhance the diversity of the library in terms of size, shape and chemical functionalities. All molecules in 110 mM DMSO-*d*_6_ stock solutions were tested for aqueous solubility (600 µM), purity and stability by ^1^H NMR spectra. Waterlogsy experiments were recorded to detect possible aggregation as LOGSY effects are characteristic of high molecular weight compounds in water [29]. When the molecules were not soluble at 600 µM, solutions were tested at 200 µM. If not soluble, the molecules were rejected from the selection. Then, to optimize the experimental time and to reduce the protein quantity required for the NMR screening experiments, fragments were pulled into mixtures of 3 to 6 molecules with minimum spectral overlap. NMR spectra of the mixtures were recorded after 3 months to confirm the absence of degradation of the compounds.

### NMR screening of the fragment library

**Figure 1:**
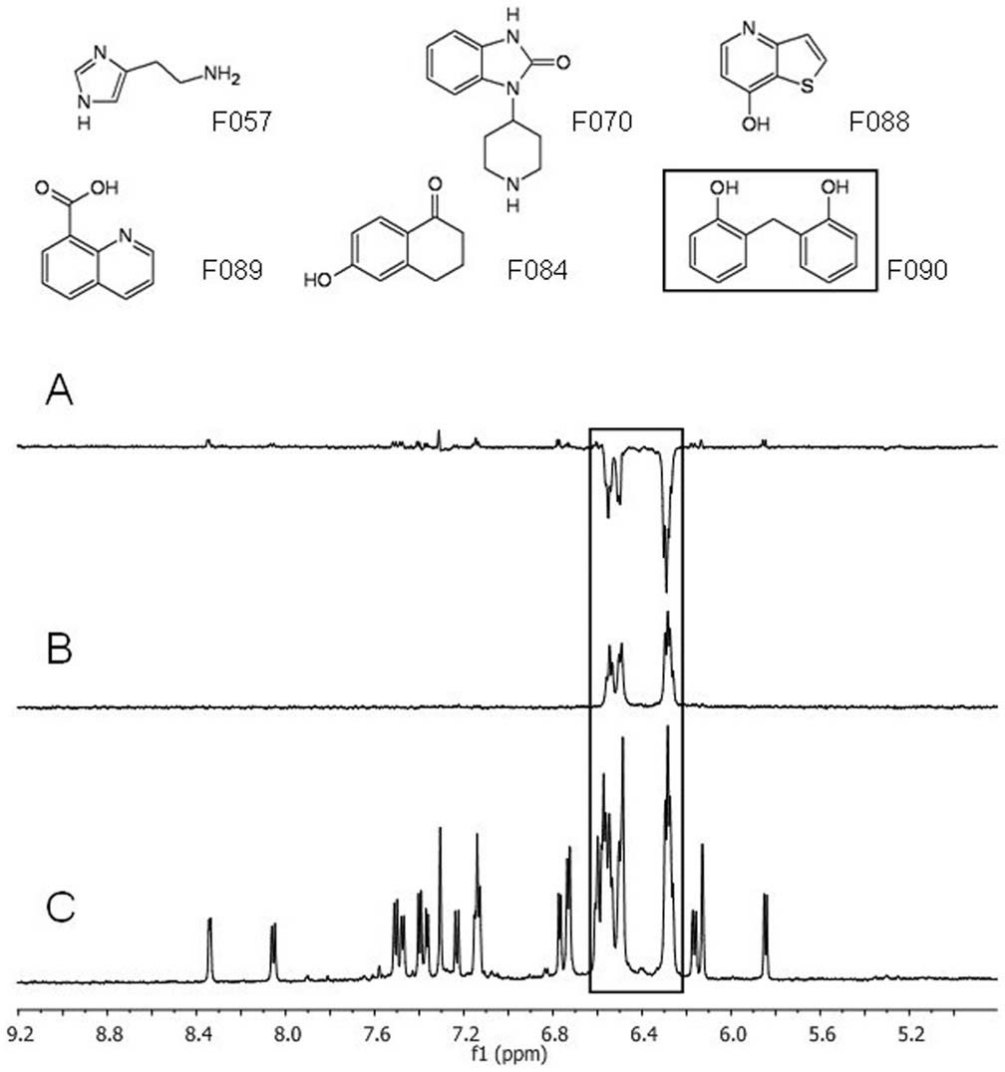
Identification of fragment molecule F090 as a ligand of PRDX5 from the NMR experiments. The Waterlogsy (**A**), STD (**B**) and normal ^1^H 1D (**C**) spectra (aromatic and ethylenic region) of a mixture of 6 fragments at a 600 µM concentration in presence of 20 μM PRDX5 are displayed. Only signals of the molecule F090 are observed in the STD experiment whereas the corresponding signals are in antiphase as compared to all other molecule signals in the Waterlogsy spectrum (framed regions).

**Figure 2:**
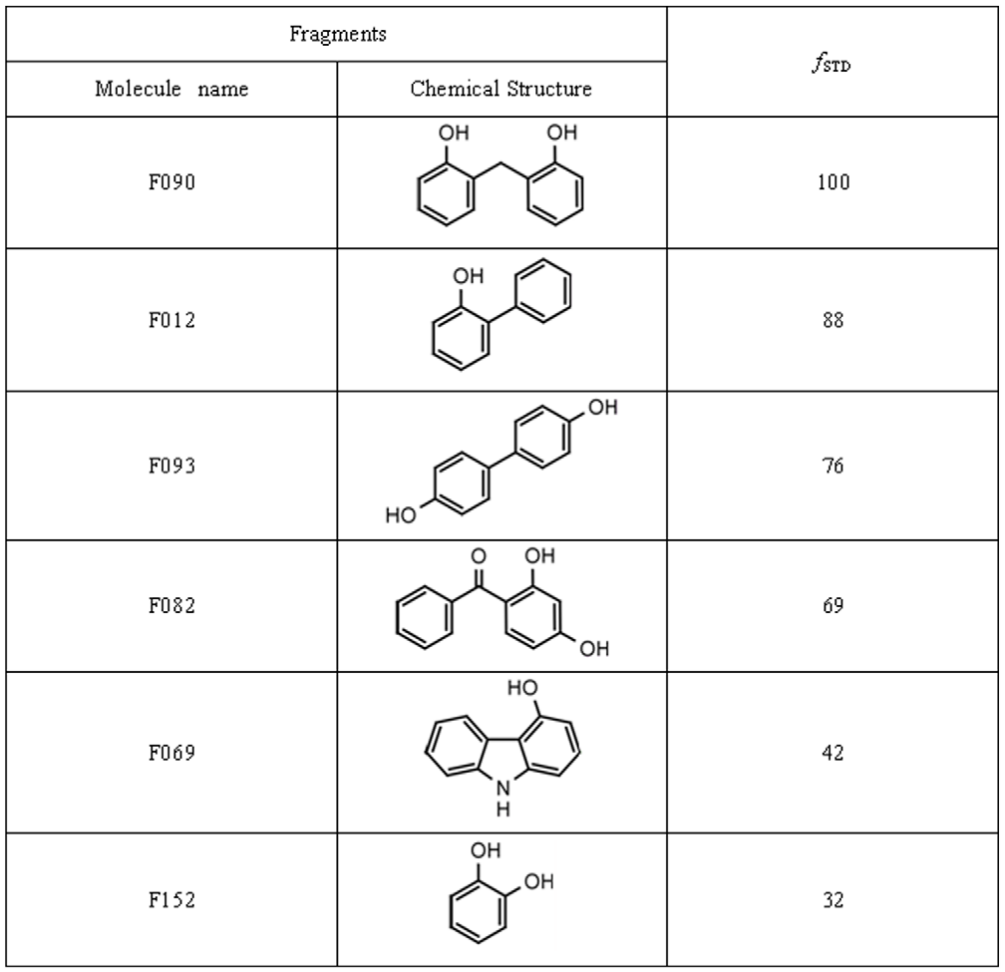
Chemical structure and STD factors of the 6 hit fragments of PRDX5 identified by NMR screening. The fragments were tested for binding by 1D NMR (STD and WaterLOGSY experiments). For each fragment, the STD factor (*f*_STD_) was determined.

The fragment library was tested on the reduced form of PRDX5 by ^1^H NMR, using both Saturation Transfer Difference (STD) [30] and Waterlogsy [31] experiments, with experimental conditions similar to those previously published [27]. The hit fragments were identified in the mixtures without the need of deconvolution. Figure 1 shows the NMR spectra recorded on one of the mixtures, and the identification of fragment F090 as a hit. Among the 200 fragments, 6 compounds gave strong STD signals as well as antiphase Waterlogsy effects in the presence of the target protein, indicating that these 6 fragments bind to PRDX5 (Figure 2). Finally, each hit fragment was tested alone in interaction with the PRDX5 protein to confirm binding.

### Binding affinity measurements

**Figure 3:**
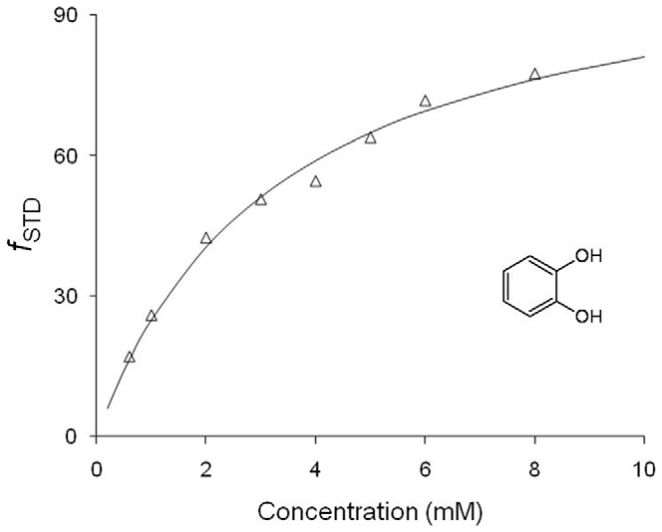
STD factors (*f*_STD_) of fragment F152 plotted as a function of ligand concentration for the measurement of the dissociation constant of the F152-PRDX5 interaction.

The binding affinities of two of the six hit fragments, F090 and F152, were determined by measurement of the STD factor variation upon titration. F152 is the smallest fragment identified here as a PRDX5 ligand, whereas F090 is the fragment that induces the strongest STD signals. Figure 3 shows the STD factor (see experimental section) as a function of the ligand concentration for F152. A dissociation constant of 3.3±0.6 mM was calculated for this fragment as described in the experimental section. For fragment F090, the binding constant K_D_ was determined to be 1.2±0.4 mM.

For the four other fragments (F012, F063, F082 and F093), the solubility in water was too low to obtain reliable measurement. However, all the hit fragments were ranked according to their affinity, using their STD factor (*f*_STD_) (Figure 2). The relative degree of saturation for the individual protons normalized to that of F090 was used to compare the STD effect. We observed that F152 and F090 respectively display the weakest and strongest STD factor. It can thus be inferred that the affinity of the six hit fragments lies in the low millimolar range, likely between 1 and 5 mM.

### Docking

**Figure 4:**
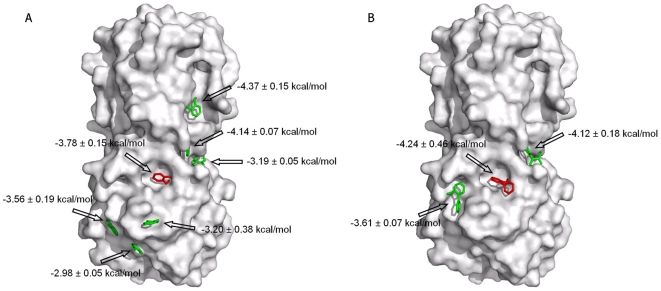
Surface representation of the PRDX5 protein (code PDB 1HD2) with the lowest-energy fragment conformer for each cluster calculated by AutoDock. (**A**) Fragment F152, (**B**) Fragment F012. The conformer located in the active site pocket of PRDX5 is colored red. Each cluster is labeled with the average binding energies.

To gain insight into the binding site of the fragments, the molecules were docked into the three-dimensional structure of the protein using the AutoDock4 program [32], [33]. AutoDock4 requires a 3D grid to be defined to represent the protein, and a Lamarckian genetic algorithm explores positions of the ligand relative to the grid. Without any knowledge of the binding sites of the fragments, the docking experiments were done using a box containing the whole protein and positions were calculated for 50 conformers. As illustrated in Figure 4, seven different binding sites are proposed by the AutoDock program for fragment F152 (Figure 4.A) and three for fragment F012 (Figure 4.B). Three to six binding sites were observed for fragments F069, F082, F090 and F093. For all the fragments, the binding energies of the different clusters are similar. As an example, the binding energies of F152 vary from −2.98 to −4.37 kcal.mol^−1^, while the AutoDock binding energies are estimated to have an error of 2.2 kcal.mol^−1^ [34]. Moreover, the lowest-energy conformers of the six hit fragments are not observed in the same binding site, as shown in Figure 4. Therefore, the docking experiments do not indicate if the six fragments detected as ligands in the NMR screening bind into the same binding site and if so, which binding site it is.

### NMR assignment of the human PRDX5 backbone resonances

To go further into the characterization of the fragment binding site, the backbone resonances of PRDX5 have been assigned. Triple-resonance NMR experiments recorded on a ^15^N/^13^C/50% ^2^H-labeled protein sample (see experimental section) were used. The deuteration was necessary due to the molecular weight of the protein (dimer of 32 kDa). The backbone assignment was confirmed with a ^15^N-edited NOESY experiment through the characteristic NOEs observed in helices and β-sheets expected from the 3D crystallographic structure (1HD2 PDB entry [25]).

### Characterization of the binding by ^15^N-HSQC experiments and docking experiments

**Figure 5:**
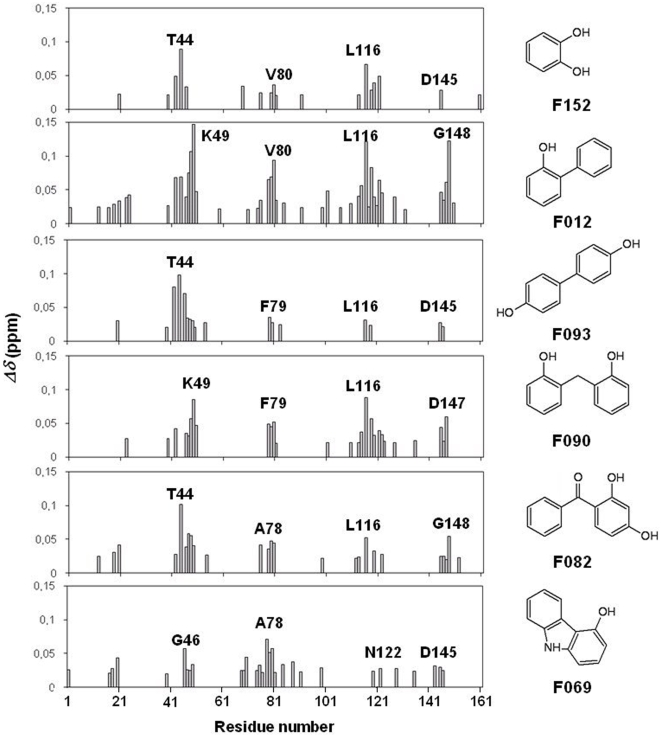
Weighted average (see experimental section) of the ^1^H^N^ and ^15^N chemical shift changes upon the addition of fragment F152, F012, F093, F090, F082 and F069, respectively. Only significant modifications (>0.02 ppm) are displayed. The residues showing the greatest changes are labeled.

To identify the amino acids involved in the protein-ligand interactions, ^15^N-HSQC (^15^N-Heteronuclear Single Quantum Correlation) spectra were acquired in absence and presence of the fragments (10-fold excess). As a result of addition of the compounds to the ^15^N-labeled protein, significant chemical shift perturbations were observed on the NMR protein spectrum (see supplementary material, Figure S1). Interestingly, the same PRDX5 residues are affected upon addition of the six fragments. Those residues belong to the N-terminal part of helix 2 (residues 44 to 49), the N-terminal of helix 3 (residues 78 to 80), the loop connecting helix 4 to sheet 8 (residues 116 to 120) and the turn preceding the C-terminal helix (residues 145 to 148) (Figure 5), and are all located in the active site region. The chemical shift perturbations mapped on the PRDX5 3D structure clearly show that the fragments bind to the PRDX5 catalytic site, in close proximity to the cysteine residue C47 (Figure S2).

**Figure 6:**
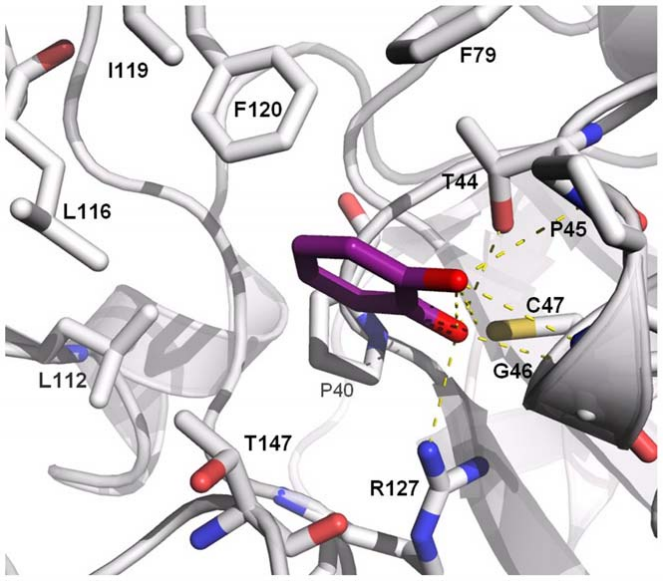
Binding mode of fragment F152 into the PRDX5 active site. The position of F152 (in magenta) was calculated with the program AutoDock [32], [33]. The residues involved in the active site pocket are labeled and displayed with sticks. Oxygen atoms, nitrogen atoms and sulfur atom are colored in red, blue and yellow, respectively.

As reported previously [35], the knowledge of the ligand binding site potentially improves the accuracy of the docking calculations by minimizing the grid volume in the AutoDock program. Here, the chemical shift changes observed in the ^15^N-HSQC experiments were used to define the docking box. Figure 6 shows fragment F152 docked against PRDX5 receptor, using a box centered on the active site. The 50 structures of F152 were well superimposed on one cluster into the active site, with a rmsd of 0.42 Å. By comparison, for the blind docking, only 16 positions out of the 50 docked structures were observed in the active site, with a rmsd of 1.09 Å. The docking results indicate that fragment F152 docks near the residue T44, in agreement with the NMR experiments (Figure 5), and highlight the role of the two hydroxyl function of F152 that point to catalytic cysteine C47 (Figure 6). Interestingly, the six fragments shown to bind PRDX5 from the 200-fragment library all share a hydroxyl function (see Figure 5). For all these fragments, a similar feature is observed from the docking calculations, with the hydroxyl function of the fragments oriented towards the cysteine C47. These hydroxyl functions might play a role of a molecular anchor that hangs the ligand to the PRDX5 protein.

### Binding mode analysis of the fragments using fragment derivatives

**Figure 7:**
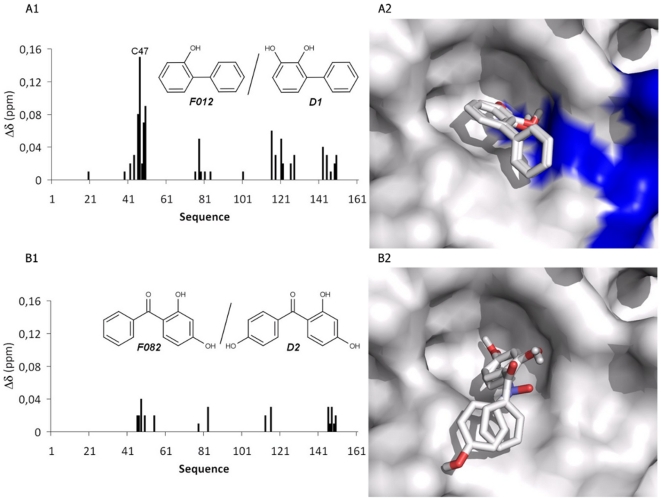
Comparison of the NMR perturbations between fragments and derivatives. (**A**) Comparison between fragment F012 and its derivative (molecule D1), (**B**) between fragment F082 and its derivative (molecule D2). (**1**) Difference of the chemical shift perturbations observed between the fragments and their derivatives. (**2**) Visualization of the binding modes. The significant NMR perturbations are mapped in the surface of the PRDX5 structure using blue color.

**Figure 8:**
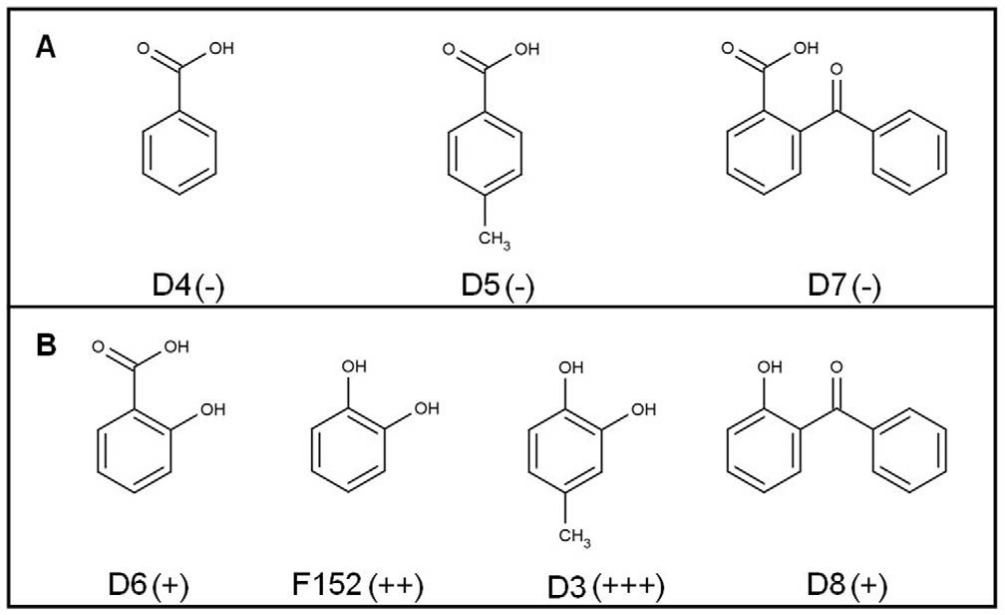
Binding experiments on benzoate and derivatives. (**A**) No binding or very weak binding detected by NMR experiments. (**B**) Addition of hydroxyl function, these fragments bind to the PRDX5 protein. The relative affinity of the fragments derivatives is indicated with (+) signs. (+) indicated a correct ligand and (+++) a very good ligand.

To confirm the binding modes of the hit fragments, we have studied the interactions of PRDX5 with homologues of fragments F012, F082 and F152. In the derivative of F012 (compound D1), a hydroxyl function is added in close proximity to the hydroxyl involved in the interaction with the active site polar region, whereas in the derivative of F082 (compound D2), the hydroxyl function is added on the second aromatic ring (Figure 7). The derivative of F152 was the 4-methyl catechol (compound D3, represented in Figure 8). According to the NMR data (ligand-observed as well as protein-observed experiments), compounds D1, D2 and D3 all bind to the PRDX5 protein, in the same region as the initial fragments. As illustrated in Figure 7, significant chemical shift changes are observed when fragment F012 is compared to compound D1, with the most significant variations found for residues located around the catalytic cysteine C47 (residues G46, C47, K49 and T50). This indicates that the additional hydroxyl function is oriented towards those residues, in agreement with the binding mode observed for F152. By contrast, the chemical shifts induced by fragment F082 and its derivative (compound D2) are very similar, with differences smaller than 0.04 ppm (Figure 7). Thus, the addition of the hydroxyl function in molecule D2 does not strongly modify the binding of the fragment, showing that the additional oxygen does not play a crucial role in the interaction with the protein. This is in agreement with a binding mode where the hydroxyl function located near the carbonyl of F082 is oriented towards the catalytic cysteine. The binding mode of F152 is corroborated as well by the binding of the 4-methyl catechol (compound D3), where the methyl attached to the aromatic ring does not prevent the binding of the molecule and interacts with the hydrophobic region of the active site.

### Binding experiments on benzoate and derivatives

In the crystal structure of PRDX5, a benzoate (compound D4) was found in the active-site pocket of the protein [19], [25]. NMR experiments (STD, Waterlogsy and HSQC) were thus recorded to study the benzoate-PRDX5 interaction in solution. The three experiments indicate that the benzoate is a very weak ligand in solution since only very weak STD signals as well as small chemical shift variations in the HSQC spectrum were observed, by comparison to the hit fragments. To go further, we have also analyzed the interactions of benzoate derivatives with the PRDX5 protein (Figure 8). First, a methyl group was added to the benzoate (Figure 8.A compound D5) but no interaction was detected by NMR. Then a hydroxyl function was added to the benzoate and this fragment (D6) binds to the protein. By substituting the benzoate carboxyl function for a hydroxyl function, we obtained the catechol (F152), which binds to the protein as previously described. The addition of the methyl function to the catechol (compound D3) does not disturb the interaction. Instead, it seems to increase the affinity to the PRDX5 protein, according to the NMR experiments (*f*_STD_ = 32% and 66% for fragment F152 and compound D3, respectively). We also tested an aromatic derivative of the benzoate (compound D7). No interaction was detected, while substitution of the carboxyl function for a hydroxyl function leads to a weak ligand (compound D8). As illustrated in Figure 8, the NMR data show that only the molecules with a hydroxyl function bind efficiently to the PRDX5 protein in our experimental conditions.

### Inhibition of thioredoxin peroxidase activity

Inhibition of thioredoxin peroxidase activity was assessed with fragments F012, F069, F082, F090, F093 and F152, and with the derivative of F152, compound D3. Inhibition measurements were not possible with fragments F090 and F093 due to insufficient solubility in the assay, and with fragments F069 and F082 (absorbance at 340 nm). Fragment F012 had no inhibitory activity (0–600 µM). Fragment F152 and its derivative compound D3 were shown to inhibit the thioredoxin peroxidase activity of PRDX5 with IC_50_ values of 105±8.5 µM and 26±2.2 µM, respectively (Figure S4).

## Discussion

Here we report a study that focuses on the interactions of small molecules with human PRDX5. To date, few inhibitors have been discovered for the PRDX family and the only ligand of PRDX5 is the benzoate anion, which was observed in the active site of the crystal structure of the enzyme [25]. However, to our knowledge, no data was available about the dissociation constant or inhibitory role of this compound. To identify ligands of the enzyme, we applied the fragment-based methodology [1], [2], [4], [8], [9] on the PRDX5 protein, using our in-house fragment library of 200 molecules. The advantage of screening molecular fragments rather than drug-sized molecules is that a dramatically larger portion of chemical structure space is explored with a smaller number of compounds. Since the fragments are small and much simpler than drug-like molecules, the fragment methodology enables to discover molecules which bind more efficiently to a small region of the protein [6], [36].

In the study presented here, the interactions of the fragments with PRDX5 were analyzed using ligand-observed NMR methods (STD [30] and Waterlogsy [31]) (Figure 1). Six fragments were shown to bind the PRDX5 protein among the 200-fragment library (Figure 2). The six hit fragments were ranked according to their affinity for PRDX5, using the STD factors (Figure 2 and Figure 3). The binding affinity was determined for two of the six ligands, F152 and F090. F152 displays the weakest STD factor and binds with a 3.3±0.6 mM affinity to PRDX5, whereas F090 has the strongest STD factor and a 1.2±0.4 mM affinity for the protein. Overall, the affinity of the 6 hit fragments should fall within a 1–5 mM range.

To characterize the binding site of the fragments, blind docking calculations were performed. In this case, the entire protein surface of the protein is scanned, and scoring functions enable to identify the binding site. However, as previously reported, it may be challenging to identify the ligand binding site when the binding energies of the different positions are similar [35]. Here, for each fragment, more than three different binding sites were found for the 50 positions docked into the 3D structure of PRDX5 (Figure 4). The binding energies of the different clusters are clearly not an efficient metric to distinguish the ligand binding site since the binding energy difference falls within the error of the calculations (2.2 kcal.mol^−1^) [34]. Therefore, to discriminate the fragment binding site, we used protein-observed NMR experiments. The PRDX5 chemical shift changes observed upon fragment addition clearly demonstrate that the six fragments bind to the protein catalytic pocket near the cysteine residue C47 (Figures 3, 4 and Figures S1 and S2). These results show that the correct position of the fragment is not necessarily the lowest-energy conformer of the docking calculations (Figure 4).

Interestingly, all the six hit fragments share the presence of one hydroxyl function (see Figure 5). The benzoate anion observed in the active site of the PRDX5 crystal structure [25] is not reported as a good PRDX5 ligand in our NMR study. Such observations are likely due to the difference between the solution state as compared to the highly-ordered crystal state. The analysis of benzoate derivatives confirms that the hydroxyl function is an important feature for the fragments to bind PRDX5 in our experimental conditions (see Figure 8). To better understand the role of these hydroxyl functions in the fragment-PRDX5 complexes, binding modes of the fragments were calculated from docking experiments where the NMR data were used to localize the fragment binding site. As illustrated in Figure 6, the active site pocket of PRDX5 is highly dissymmetric with one side strongly hydrophobic (P40, L112, L116, I119, F120 from chain A and F79 from chain B) whereas the opposite side is polar due to residues T147, R127, G46 and T44. In the fragment-PRDX5 complexes, the aromatic part of the fragments interacts with the hydrophobic side chains of the pocket, whereas the hydroxyl function is oriented towards the catalytic cysteine and is involved in polar interactions with main chain or side chain atoms of residues G41, T44, P45, G46, C47 and R127.

Figure 6 illustrates the binding mode of fragment F152, as suggested by the docking calculations. One oxygen of F152 makes contacts with T44 (distance 2.91 Å), C47 (3.18 Å) and R127 (2.96 Å) whereas the second oxygen interacts with P45 (3.80 Å) and G46 (2.98 Å). The aromatic ring lies in the hydrophobic pocket and makes hydrophobic contacts with L116, I119, F120, and F79. It was interesting to observe the structural similarities of fragment F152 and the corresponding X-ray conformation of the benzoate [25]. The two structures superimpose pretty well (0.97 Å rmsd) and the two functions, dihydroxyl for the catechol and carboxylic for the benzoate, lie in the same site (Figure S3). In the two structures, the binding motif is represented by two oxygen atoms that point to the C47 residue. Interestingly, such interactions roughly mimic the interactions of a peroxidatic substrate [19], [25], even if the O-O distance is here much greater (2.75 Å for the catechol and 2.24 Å for the benzoate as compared to 1.54 Å for a peroxidatic substrate).

The binding mode proposed by the combination of NMR experiments and docking calculations was confirmed by the study of derivatives of F012, F082 and F152 (Figure 7). The comparison of the ligand induced chemical shift changes between the ligands and their derivatives demonstrate that the oxygen atoms point towards the catalytic cysteine C47. The addition of a methyl group to F152 (compound D3, Figure 8) corroborates the results, and the interaction is optimized by increasing the hydrophobicity of the aromatic part of the molecule. As illustrated in Figure 7, the molecules with two aromatic rings such as F012 and F082 do not entirely fit the PRDX5 active site, which is a rather small pocket (about 9 Å diameter). This could explain the low hit rate (3%) observed for PRDX5, when compared to other proteins screened against the same fragment library (unpublished data) and may suggest that the PRDX5 protein is not highly druggable [37].

To assess the quality of the hit fragments as starting points for inhibitor design, the ligand efficiencies of F090 (bis-(2-hydroxyphenyl)-methan) and F152 (catechol) were determined. Ligand efficiency is the free energy of binding divided by the number of heavy atoms [5], [6], and is commonly used to predict if a ligand can potentially be elaborated into a good inhibitor, and to rank hit compounds in order to choose the best lead molecule. The ligand efficiencies of F090 and F152 were calculated to be 0.42 kcal.mol^−1^ and 0.26 kcal.mol^−1^ respectively. In a recent study where 18 highly optimized inhibitors were deconstructed, a nearly linear relationship was observed between molecular weight and binding efficiency [38]. It was extrapolated that, considering that the LE is kept constant during the fragment evolution, a good starting point should exhibit a LE of 0.3 in order to yield a molecule of 500 g.mol^−1^ with a IC_50_<10 nM [39]. These data thus indicate that fragment F152 represents a very interesting starting point for the design of a PRDX5 inhibitor. This hypothesis was confirmed by an enzymatic assay showing that F152 significantly inhibits the enzymatic activity of PRDX5, with an IC_50_ value of 105±8.5 µM.

The docking calculations suggest that improvement of the ligand affinities could be achieved through optimization of the polar contacts with functional group of T144, R139 and T147 (both side chain and main chain). Hydrophobic interaction should also be strongly strengthened through contacts with L116 and L112. This hypothesis was confirmed by the analysis of a derivative of F152, compound D3, which corresponds to the catechol with an additional methyl moiety. This molecule displays a stronger STD signal than F152 (*f*_STD_ = 32% and 66% for F152 and compound D3, respectively). These NMR data were corroborated by the results of a biochemical assay showing that the inhibitory activity of F152 is increased by the addition of the methyl moiety on the phenyl ring (IC_50_ = 105±8.5 µM and 26±2.2 µM for F152 and compound D3, respectively). Another strategy for optimization of the fragments could be to target both the active site pocket and an adjacent binding site highlighted by docking calculation. This second pocket is located at the interface of the PRDX5 dimer. Here, molecules should be designed to interact with E83 and S48 side chains.

**Figure 9:**
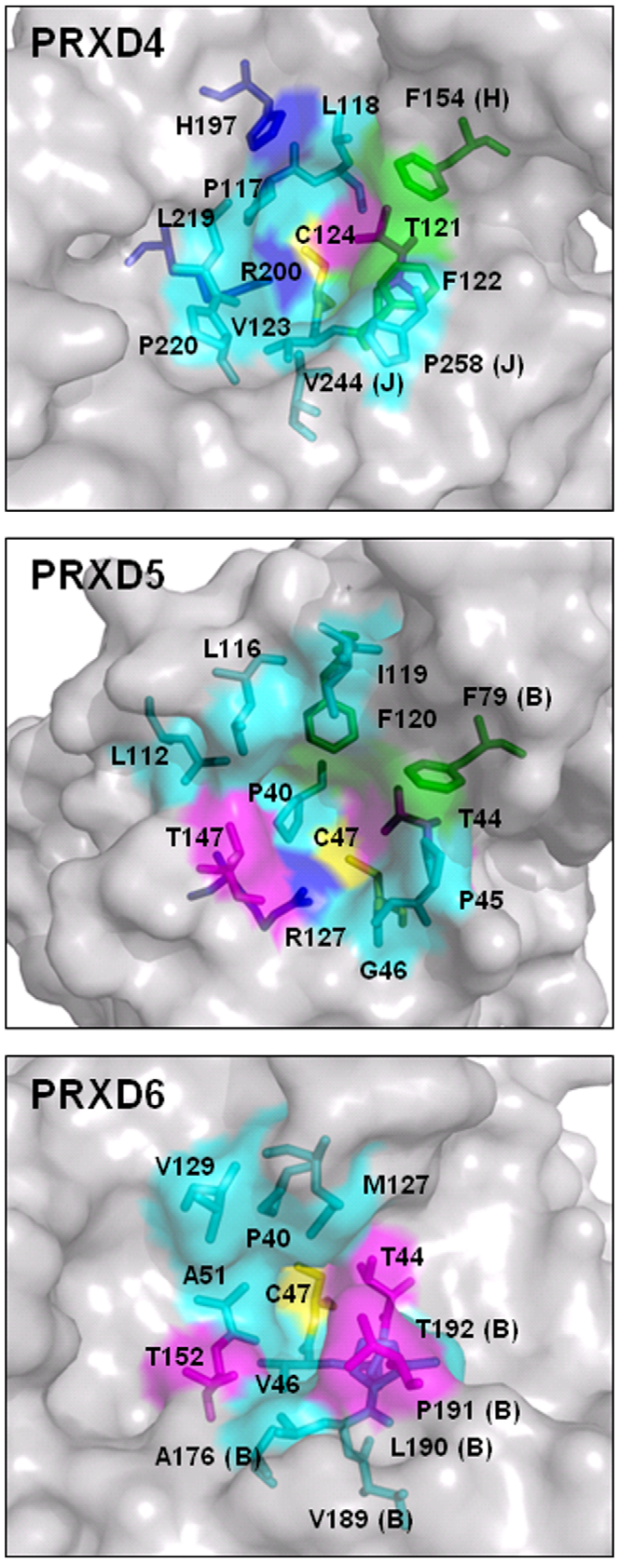
Active site pockets of the three human PRDX groups. The peroxidatic cysteine is colored in yellow. Aromatic residues are colored in green, other hydrophobic residues are colored in cyan and polar residues (Thr, Met) are colored in magenta. Residues that are located on a protein chain different from the cysteine one are labeled with the chain name. (PRDX4 is a decamer and PRDX5 and PRDX6 are dimers). The figure was generated using the PDB structures 2PN8 (PRDX4), 1HD2 (PRDX5) and 1PRX (PRDX6).

These small PRDX5 ligands thus provide the first basis for the design of non covalent PRDX inhibitors. However, will it be possible to design a selective inhibitor for the different PRDXs? Despite the broad sequence diversity represented among the different Peroxiredoxin subfamilies, the geometry of the active site region is rather conserved, with a proline residue (P40), a threonine residue (T44) and an arginine residue (R127) in van der Waals contact with the peroxidatic cysteine (C47). Yet, the three-dimensional structures of PRDX4 (2-Cys Peroxiredoxin, 2PN8 PDB code), PRDX5 (Atypical 2-Cys Peroxiredoxin, 1HD2 PDB code) and PRDX6 (1-Cys Peroxiredoxin, 1PRX PDB code) show that the quaternary structure dramatically varies among the subfamilies. As illustrated in Figure 9, the shapes of the PRDX active sites are modulated by the quaternary structures. PRDX4 and PRDX5 active sites look rather similar whereas the active site of PRDX6 displays very characteristic features, with a narrow pocket of ∼4 Å diameter and ∼7 Å depth [16]. The bottom of the PRDX6 cavity is largely hydrophobic while the entry of the pocket is much more polar due to residues T152 (chain A) and T192 (chain B). By contrast, the active site pocket of PRDX5 is more cylindrical and less deep. The pocket is dissymmetric with one hydrophobic side facing a polar region (see above). The overall shape of the PRDX4 cavity appears to be an oval pocket, with a hydrophobic patch interrupted by residue H197 while residue T147 of PRDX5 is replaced in PRDX4 by a hydrophobic residue. Therefore, in spite of the general idea that the PRDXs share common active site features, we show here that the pockets differ, both in their conformation and accessible surface properties. These significant differences could be used to guide the design of selective inhibitors. If the molecule F152 is not complex enough to be a selective fragment, its modification by addition of selected chemical functions to its phenyl moiety should provide potency as well as selectivity.

In conclusion, this study reports molecules demonstrated to bind the PRDX5 active site by NMR experiments and enzymatic assays. The binding mode of these weak ligands was characterized by a combination of NMR data and docking calculations and was confirmed using fragment homologues, highlighting the role of hydroxyl functions. One fragment, the catechol, was shown to possess a high ligand efficiency and to exhibit inhibitory activity against PRXD5, indicating that this molecule is an excellent starting point for the development of potential PRDX inhibitors.

## Materials and Methods

### Fragment library

The compounds of the library were chosen from Aldrich or Acros online catalogs with SHAPES-like criteria [40]–[42] and had to fit the rule of three [28]. Aqueous solubility was checked for all compounds by recording ^1^H 1D NMR spectrum and Waterlogsy spectrum [31], [43]. 110 mM stock solutions of the library compounds were prepared in DMSO-*d*_6_ and conserved at −20°C. ^1^H 1D NMR spectrum were recorded to check that no degradation occurs over 3 months. Compounds were mixed by 3 to 6 to decrease the NMR experimental time as well as the protein quantity. Criteria for the compound selection for the mixtures were the absence of overlapping resonances in the 1D NMR spectra. The library contained 200 validated fragments.

### Protein production and purification

Human PRDX5 was expressed without its mitochondrial targeting sequence as a N-terminal 6xHis-tagged protein in *E. coli* strain M15 (pRep4) [44]. *E. coli* were grown at 37°C in M9 minimal medium supplemented with thiamine (20 mM) and containing ^15^NH_4_Cl (1 g/L) to produce ^15^N-PRDX5 or ^15^NH_4_Cl (1 g/L), [^13^C_6_]-D-glucose (4 g/L) and 70∶30% (v/v) D_2_O∶H_2_O to produce ^15^N/^13^C/50% ^2^H-PRDX5. Recombinant PRDX5 was purified by affinity chromatography on a Ni^2+^-NTA column (Qiagen) as previously described [44].

### NMR screening

All spectra were acquired at 20°C with a Varian Inova 600 MHz NMR spectrometer, equipped with a standard 5 mm triple-resonance inverse probe with a *z*-axis field gradient, actively shielded, and with an autosampler robot. The NMR samples were prepared with a robot TECAN Miniprep 60. For 175 fragments, 32 NMR tubes were prepared with 20 µM of the reduced protein and 600 µM fragments. For 25 fragments (6 NMR samples) that were not soluble at 600 µM in water, the final concentration in the NMR tube was set to 200 µM. All samples contained 1 mM DTT. The concentration of DMSO-*d*_6_ did not exceed 4% in the NMR tubes. Control 1D ^1^H spectra preceded all experiments to assess the purity and stability of the fragments. NMR screening was achieved using 1D STD [30] and Waterlogsy [31] experiments. The parameters used were the same as previously described [27]. All NMR spectra were processed with the Varian VnmrJ software.

### K_D_ measurements

Dissociation constants (K_D_) were obtained for selected compounds by monitoring the STD amplification factors (*f*_STD_) as a function of ligand concentration. *f*_STD_ were derived from the equation *f*_STD_ = *I*_STD_/*I*_0_ * ([L]_tot_/[PRDX5]_tot_), where *I*_STD_ and *I_0_* are the peak integrals in the STD and 1D ^1^H experiments respectively, and [L]_tot_ and [PRDX5]_tot_ are the total concentrations of the ligand and PRDX5, respectively. K_D_ values were determined by fitting the plot of [L]_tot_ vs. *f*_STD_ as previously published [27].

### Docking

AutoDock 4.01 [32], [33] with the AutoDockTools graphical interface was used to simulate 50 different binding conformations for each PRDX5 ligand. Grid maps were generated with 0.375 Å spacing and set to encompass the residues perturbed upon fragment addition. The docking calculations were then performed using the Lamarckian genetic algorithm (LGA) for ligand conformational searching. The population size was set to 150 and the number of energy evaluations was 2500000. The 3D structure of PRDX5 was used (1HD2 PDB entry) and the dimer was built from the coordinates of the monomer using Makemultimer from the Expasy Proteomics server (http://expasy.org/tools/).

### Backbone resonance protein assignment

NMR samples contained 500 µM of uniformly ^15^N/^13^C/50% ^2^H labeled reduced PRDX5 in 10 mM phosphate buffer, 3 mM KCl, 140 mM NaCl pH 7.45 and 2 mM DTT. 3D HNCA, HN(CO)CA, HNCACB and CBCA(CO)NH experiments from the Varian Protein Pack were recorded at 28°C on a Varian Inova 600 MHz NMR spectrometer. A ^15^N-HSQC spectrum was collected before and after each 3D experiment to check the protein stability. A 3D ^1^H-^15^N NOESY-HSQC experiment was also recorded with a mixing-time of 150 ms. All NMR spectra were processed with NMRPIPE software [45] and analyzed using NMRView [46].

### HSQC experiments

HSQC spectra were acquired with 128 scans and 64 t_1_ increments on 550 µl of uniformly ^15^N-labeled reduced PRDX5 at 100 µM in the presence and absence of added compound. Compounds were tested at 1 mM or 2mM each and binding was analyzed by monitoring changes in the ^15^N HSQC spectra. The HSQC spectra were processed with NMRPIPE software [45] and analyzed using NMRView [46]. The chemical shift changes were calculated using the weighted average of the ^1^H and ^15^N chemical shift changes Δ_AV_ according to the equation Δ_AV_ = [(Δδ^2^_H_ + Δδ^2^_N_/25)/2]^1/2^ [47].

### Thioredoxin peroxidase activity inhibition assay

Inhibition of thioredoxin peroxidase activity was assayed using thioredoxin peroxidase assay in a 96-well plate reader essentially as described by Kim et al. [48] and Theys et al. [49]. Briefly, the spectrometric assay was performed in a 160 µl reaction mixture containing 500 µM NADPH, 4 µM recombinant *S.cerevisiae* thioredoxin, 2 µM recombinant *S.cervisiae* thioredoxin reductase, 0.6 µM human recombinant PRDX5 and increasing concentrations (0 to 6 mM) of fragments (from stock solutions in DMSO) in PBS 0.1M (pH 7.4). The reaction was initiated by adding H_2_O_2_ at the final concentration of 100 µM. NADPH oxidation was monitored by following absorbance at 340 nm for 30 min at 37°C. The initial rate of reaction was calculated using the linear portion of the curve and was expressed as the amount of NADPH oxidized per min. The IC_50_ was calculated with the percentage of remaining thioredoxin peroxidase activity. Measurements were performed in quadruplicates.

### Supporting Information Available

Figures showing the chemical shift perturbations of the PRDX5 protein upon addition of fragments, identification of the binding region for the 6 hit fragments, superposition of the docked structure of fragment F152 with the X-ray structure of the benzoate, and the plot of the dose-response relationship of the inhibition of PRDX5 by F152.

## Supporting Information

Figure S1 Chemical shift perturbation of the ^15^N-HSQC spectrum of PRDX5 (80 µM) in absence (black contours) and presence of 1 mM fragment F012 (blue), F090 (red) and F082 (green). The residues that exhibit significant chemical shift perturbations are labeled according to their sequence-specific assignment.
(1.08 MB TIF)

Figure S2 Identification of the binding region of the fragments into the 3D structure of PRDX5 (1HD2 PDB entry). The four regions 1 to 4 described in the text are labeled. The chemical shift variations are mapped into the 3D structure of the protein and colored in magenta. Proline residues located near the highlighted region are colored in blue (no NMR data could be obtained due to the absence of amide proton). In the same way, unassigned residues are colored in green. The peroxidatic cysteine residue is colored in yellow. (A) fragment F012, (B) fragment F069, (C) fragment F082, (D) fragment F090, (E) fragment F093 and (F) fragment F152.
(1.86 MB TIF)

Figure S3 Superposition of the F152 fragment docked structure (magenta) with the original X-ray structure for benzoate (yellow) complexed with PRDX5. The PRDX5 surface is colored according to the electrostatic potential (red for the negative region, blue for the positive surface and yellow for the cysteine residue).
(2.94 MB TIF)

Figure S4 Dose-response relationship of the inhibition of PRDX5 by F152. The estimated IC_50_ from this plot is 105+8.5 µM.
(2.10 MB TIF)

## References

[1] Congreve M, Chessari G, Tisi D, Woodhead AJ (2008). Recent developments in fragment-based drug discovery.. J Med Chem.

[2] Hajduk PJ, Greer J (2007). A decade of fragment-based drug design: strategic advances and lessons learned.. Nat Rev Drug Discov.

[3] Shuker SB, Hajduk PJ, Meadows RP, Fesik SW (1996). Discovering high-affinity ligands for proteins: SAR by NMR.. Science.

[4] Erlanson DA (2006). Fragment-based lead discovery: a chemical update.. Curr Opin Biotech.

[5] Hopkins AL, Groom CR, Alex A (2004). Ligand efficiency: a useful metric for lead selection.. Drug Disc Today.

[6] Kuntz ID, Chen K, Sharp KA, Kollman PA (1999). The maximal affinity of ligands.. Proc Nat Acad Sci USA.

[7] Ciulli A, Abell C (2007). Fragment-based approaches to enzyme inhibition.. Curr Opin Biotech.

[8] Congreve M, Murray CW, Carr R, Rees DC (2007). Fragment-Based Lead Discovery.. Ann Rep Med Chem.

[9] Jhoti H, Cleasby A, Verdonk M, Williams G (2007). Fragment-based screening using X-ray crystallography and NMR spectroscopy.. Curr Opin Chem Biol.

[10] Ni F (1994). Recent Developments in Transferred Noe Methods.. Progress in NMR.

[11] Pellecchia M, Bertini I, Cowburn D, Dalvit C, Giralt E (2008). Perspectives on NMR in drug discovery: a technique comes of age.. Nat Rev Drug Discov.

[12] Pintacuda G, John M, Su XC, Otting G (2007). NMR structure determination of protein-ligand complexes by lanthanide labeling.. Accounts Chem Res.

[13] Cioffi M, Hunter CA, Packer MJ, Spitaleri A (2008). Determination of protein-ligand binding modes using complexation-induced changes in H-1 NMR chemical shift.. J Med Chem.

[14] McCoy MA, Wyss DF (2000). Alignment of weakly interacting molecules to protein surfaces using simulations of chemical shift perturbations.. J Biomol NMR.

[15] Stark J, Powers R (2008). Rapid protein-ligand costructures using chemical shift perturbations.. Journal of the American Chemical Society.

[16] Choi HJ, Kang SW, Yang CH, Rhee SG, Ryu SE (1998). Crystal structure of a novel human peroxidase enzyme at 2.0 angstrom resolution.. Nature Struct Biol.

[17] Ellis HR, Poole LB (1997). Novel application of 7-chloro-4-nitrobenzo-2-oxa-1,3-diazole to identify cysteine sulfenic acid in the AhpC component of alkyl hydroperoxide reductase.. Biochemistry.

[18] Flohé L, Harris JR, Flohé, Harris (2007). Peroxiredoxin systems.. Subcellular Biochemistry.

[19] Karplus PA, Hall A, Flohé, Harris (2007). Peroxiredoxin systems.. Subcellular Biochemistry.

[20] Knoops B, Loumaye E, van der Eecken V, Flohé, Harris (2007). Peroxiredoxin systems.. Subcellular Biochemistry.

[21] Poole LB, Flohé, Harris (2007). Peroxiredoxin systems.. Subcellular Biochemistry.

[22] Trujillo M, Ferrer-Sueta G, Thomson L, Flohe L, Radi R, Flohé, Harris (2007). Peroxiredoxin systems.. Subcellular Biochemistry.

[23] Ogusucu R, Rettori D, Munhoz DC, Netto LES, Augusto O (2007). Reactions of yeast thioredoxin peroxidases I and II with hydrogen peroxide and peroxynitrite: Rate constants by competitive kinetics.. Free Radic Biol Med.

[24] Rhee SG, Chae HZ, Kim K (2005). Peroxiredoxins: A historical overview and speculative preview of novel mechanisms and emerging concepts in cell signaling.. Free Radic Biol Med.

[25] Declercq JP, Evrard C, Clippe A, Vander Stricht D, Bernard A (2001). Crystal structure of human peroxiredoxin 5, a novel type of mammalian peroxiredoxin at 1.5 angstrom resolution.. J Mol Biol.

[26] Liu G, Botting CH, Evans KM, Walton JA, Xu G (2010). Optimisation of conoidin A, a peroxiredoxin inhibitor.. ChemMedChem.

[27] Bretonnet AS, Jochum A, Walker O, Krimm I, Goekjian P (2007). NMR screening applied to the fragment-based generation of inhibitors of creatine kinase exploiting a new interaction proximate to the ATP binding site.. J Med Chem.

[28] Congreve M, Carr R, Murray C, Jhoti H (2003). A rule of three for fragment-based lead discovery?. Drug Disc Today.

[29] Dalvit C, Fogliatto G, Stewart A, Veronesi M, Stockman B (2001). WaterLOGSY as a method for primary NMR screening: Practical aspects and range of applicability.. Journal of Biomolecular Nmr.

[30] Mayer M, Meyer B (1999). Characterization of ligand binding by saturation transfer difference NMR spectroscopy.. Angew Chem Int Ed.

[31] Dalvit C, Pevarello P, Tato M, Veronesi M, Vulpetti A (2000). Identification of compounds with binding affinity to proteins via magnetization transfer from bulk water.. J Biomol NMR.

[32] Huey R, Morris GM, Olson AJ, Goodsell DS (2007). A semiempirical free energy force field with charge-based desolvation.. J Comput Chem.

[33] Morris GM, Goodsell DS, Halliday RS, Huey R, Hart WE (1998). Automated docking using a Lamarckian genetic algorithm and an empirical binding free energy function.. J Comput Chem.

[34] Rosenfeld RJ, Goodsell DS, Musah RA, Morris GM, Goodin DB (2003). Automated docking of ligands to an artificial active site: augmenting crystallographic analysis with computer modeling.. J Comput Aid Mol Des.

[35] Stark J, Powers R (2008). Rapid protein-ligand costructures using chemical shift perturbations.. J Am Chem Soc.

[36] Reynolds CH, Tounge BA, Bembenek SD (2008). Ligand binding efficiency: Trends, physical basis, and implications.. J Med Chem.

[37] Hajduk PJ, Huth JR, Fesik SW (2005). Druggability indices for protein targets derived from NMR-based screening data.. J Med Chem.

[38] Hajduk PJ (2006). Fragment-based drug design: How big is too big?. J Med Chem.

[39] Verdonk ML, Rees DC (2008). Group efficiency: A guideline for hits-to-leads chemistry.. Chem Med Chem.

[40] Bemis GW, Murcko MA (1996). The properties of known drugs. 1. Molecular frameworks.. J Med Chem.

[41] Bemis GW, Murcko MA (1999). Properties of known drugs. 2. Side chains.. J Med Chem.

[42] Fejzo J, Lepre CA, Peng JW, Bemis GW, Ajay (1999). The SHAPES strategy: an NMR-based approach for lead generation in drug discovery.. Chem & Biol.

[43] Dalvit C, Fogliatto G, Stewart A, Veronesi M, Stockman B (2001). WaterLOGSY as a method for primary NMR screening: Practical aspects and range of applicability.. J Biomol NMR.

[44] Declercq JP, Evrard C, Clippe A, Stricht DV, Bernard A (2001). Crystal structure of human peroxiredoxin 5, a novel type of mammalian peroxiredoxin at 1.5 A resolution.. J Mol Biol.

[45] Delaglio F, Grzesiek S, Vuister GW, Zhu G, Pfeifer J (1995). NMRPipe: a multidimensional spectral processing system based on UNIX pipes.. J Biomol NMR.

[46] Johnson BA, Blevins RA (1994). Nmr View - a Computer-Program for the Visualization and Analysis of Nmr Data.. J Biomol NMR.

[47] Grzesiek S, Stahl SJ, Wingfield PT, Bax A (1996). The CD4 determinant for downregulation by HIV-1 Nef directly binds to Nef. Mapping of the Nef binding surface by NMR.. Biochemistry.

[48] Kim JA, Park S, Kim K, Rhee SG, Kang SW (2005). Activity assay of mammalian 2-cys peroxiredoxins using yeast thioredoxin reductase system.. Anal Biochem.

[49] Theys N, Clippe A, Bouckenooghe T, Reusens B, Remacle C (2009). Early low protein diet aggravates unbalance between antioxidant enzymes leading to islet dysfunction.. PLoS One.

