# Supplementary figures and images for: Discovery of Fragment Molecules That Bind the Human Peroxiredoxin 5 Active Site
Source: PLoS One. 2010 Mar 17;5(3):e9744. doi: 10.1371/journal.pone.0009744 (PMC2840032; doi:10.1371/journal.pone.0009744)

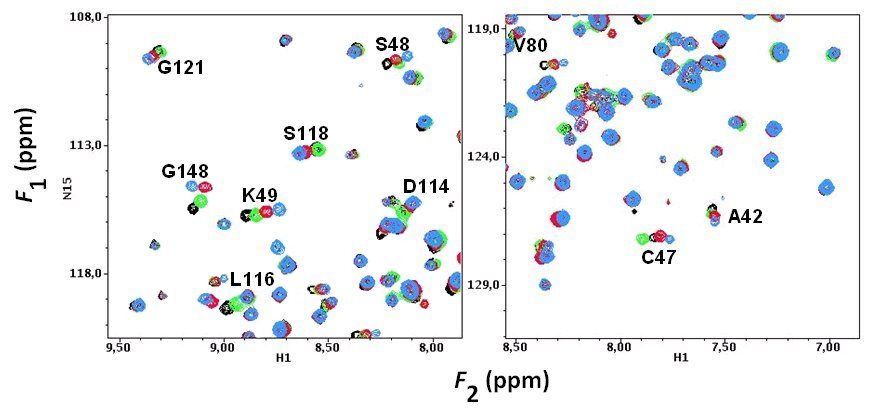

Supplement: Figure S1 — Chemical shift perturbation of the 15N-HSQC spectrum of PRDX5 (80 µM) in absence (black contours) and presence of 1 mM fragment F012 (blue), F090 (red) and F082 (green). The residues that exhibit significant chemical shift perturbations are labeled according to their sequence-specific assignment. (1.08 MB TIF) [file pone.0009744.s001.tif]

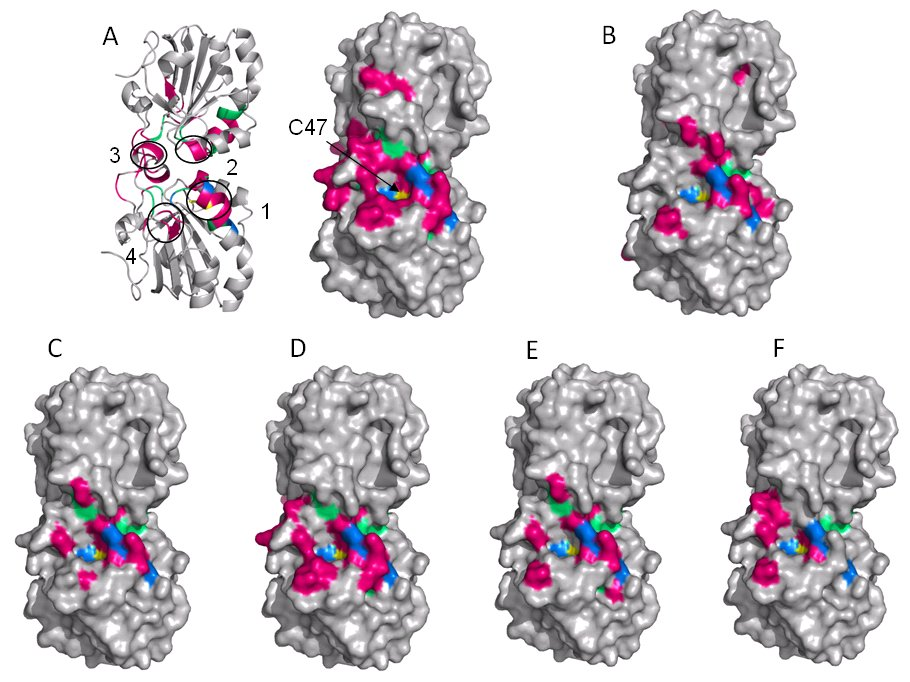

Supplement: Figure S2 — Identification of the binding region of the fragments into the 3D structure of PRDX5 (1HD2 PDB entry). The four regions 1 to 4 described in the text are labeled. The chemical shift variations are mapped into the 3D structure of the protein and colored in magenta. Proline residues located near the highlighted region are colored in blue (no NMR data could be obtained due to the absence of amide proton). In the same way, unassigned residues are colored in green. The peroxidatic cysteine residue is colored in yellow. (A) fragment F012, (B) fragment F069, (C) fragment F082, (D) fragment F090, (E) fragment F093 and (F) fragment F152. (1.86 MB TIF) [file pone.0009744.s002.tif]

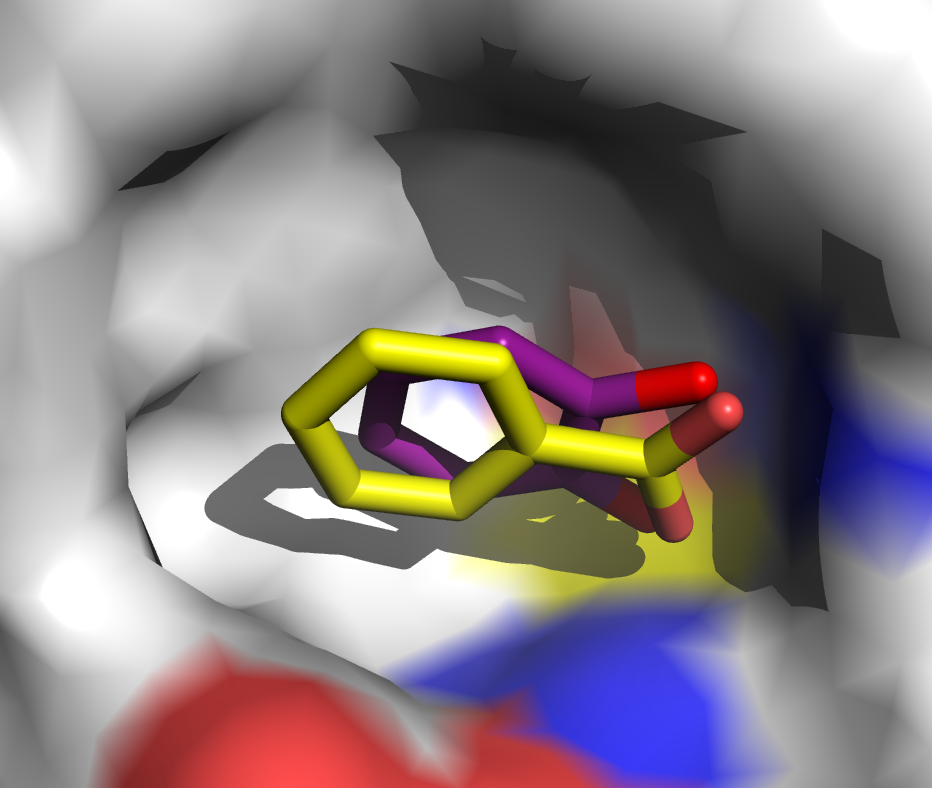

Supplement: Figure S3 — Superposition of the F152 fragment docked structure (magenta) with the original X-ray structure for benzoate (yellow) complexed with PRDX5. The PRDX5 surface is colored according to the electrostatic potential (red for the negative region, blue for the positive surface and yellow for the cysteine residue). (2.94 MB TIF) [file pone.0009744.s003.tif]

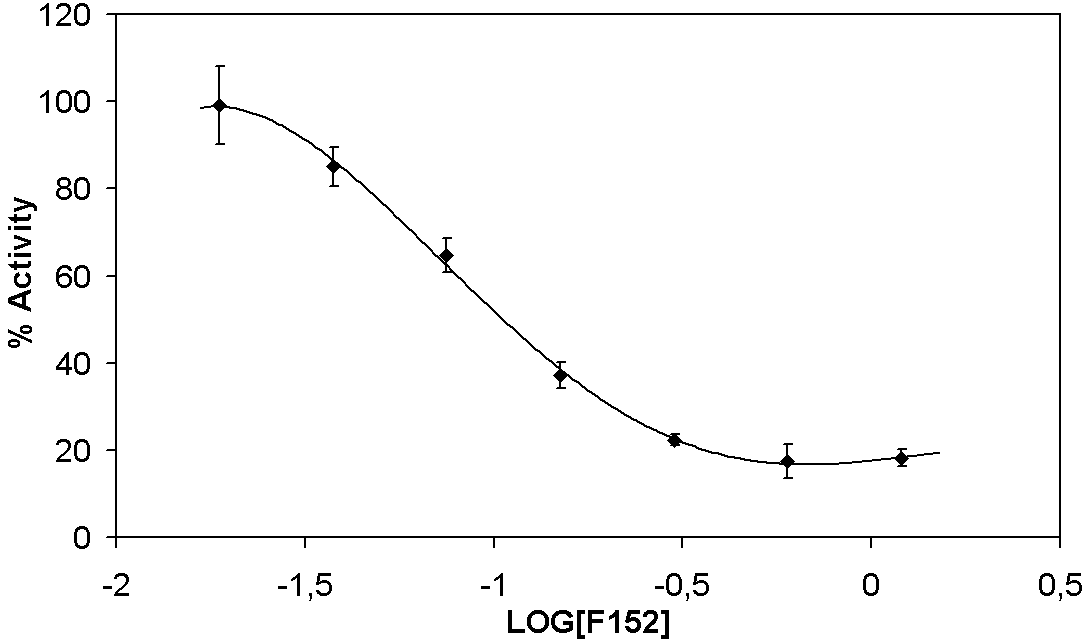

Supplement: Figure S4 — Dose-response relationship of the inhibition of PRDX5 by F152. The estimated IC50 from this plot is 105+8.5 µM. (2.10 MB TIF) [file pone.0009744.s004.tif]
